# Associated factors, nonlinear risk patterns and effect heterogeneity of severe injuries in recreational skiers and snowboarders

**DOI:** 10.3389/fpubh.2026.1814705

**Published:** 2026-04-13

**Authors:** Xiaoyu Ling, Lijun Long, Dongting Jiang, Xiaoyu Han, Xinping Song, Shuang Qin, Guole Jiang

**Affiliations:** 1 School of Physical Education, Southwest University, Chongqing, China; 2 Sports Coaching College, Beijing Sport University, Beijing, China; 3 College of Physical Education, Hunan Normal University, Changsha, China; 4 School of Athletic Performance, Shanghai University of Sport, Shanghai, China; 5 School of Coaching, Shanghai University of Sport, Shanghai, China; 6 College of Nursing, Zhangjiakou University, Zhangjiakou City, China; 7 Basic Education College, National University of Defense Technology, Changsha, China; 8 School of Acupuncture-Moxibustion, Tuina and Rehabilitation, Hunan University of Chinese Medicine, Changsha, China; 9 School of Sports Medicine and Rehabilitation, Beijing Sport University, Beijing, China

**Keywords:** injury severity, protective equipment, risk-taking behavior, snow sports, warm-up

## Abstract

**Background:**

Severe injuries in recreational alpine skiing and snowboarding impose disproportionate clinical and societal burden. Evidence on modifiable countermeasures beyond helmets remains fragmented and may vary across risk profiles and exposure conditions. This study aimed to identify factors associated with severe injuries among recreational skiers and snowboarders, and to examine nonlinear dose–response relationships and effect modification by preventive practices.

**Methods:**

We conducted a retrospective cross-sectional injury-severity study of injured adult skiers and snowboarders treated at resort medical clinics and emergency departments at two ski resorts in Zhangjiakou, China, across three winter seasons from 2021 to 2024. Severe injury was defined as an injury severity score (ISS) above 15. We modeled severe injury conditional on injury using Firth-penalized logistic regression. Restricted cubic splines were applied for age, body mass index, temperature, and snow depth to assess nonlinear associations. Prespecified interaction blocks were tested using joint Wald tests with false discovery rate control, and scenario-standardized impact metrics were estimated with bootstrap uncertainty.

**Results:**

Among 2,369 injured participants, 339 (14.3%) sustained severe injuries. In fully adjusted models, knee protector use (OR = 0.57, *p* = 0.005), cautious risk behavior (OR = 0.46, *p* < 0.001), and advanced skill level (OR = 0.46, *p* = 0.023) were associated with lower odds of severe injury, whereas collisions with other participants were associated with higher severity (OR = 1.51, *p* = 0.011). Dose–response analyses suggested non-linear patterns across continuous variables, with statistically significant overall evidence for snow depth. Warm-up and binding tests showed stronger protection in high-risk subgroups such as beginners and risk-takers, although scenario estimates were imprecise.

**Conclusion:**

Severe outcomes among injured snow-sport participants reflect interacting exposure intensity, environment, and modifiable practices. Findings support risk-stratified prevention and condition-aware resort safety operations.

## 1 Introduction

Recreational alpine skiing and snowboarding (collectively referred to as “snow sports”) are predictable seasonal sources of unintentional injuries that frequently require care in resort medical emergency centers and hospital emergency departments (EDs). Although many studies have described overall injury patterns, severe outcomes warrant focused attention because they account for disproportionate downstream costs, including hospital admission, operative management, prolonged rehabilitation, and the risk of persistent functional limitations, despite representing a minority of ED-treated cases (1, 2).

From a safety science perspective, severe injury in snow sports reflects a coupled system in which exposure intensity, accident mechanism, and environmental conditions interact. High-energy events such as collisions and high-speed falls represent key mechanisms for elevated injury severity, while behavioral and skill factors shape exposure intensity through speed choice, terrain selection, and control margins (3, 4). Terrain parks and half-pipes may further concentrate higher-energy mechanisms and more severe outcomes (5). Meanwhile, environmental conditions (e.g., temperature and snow depth) plausibly influence control, friction, and impact properties, yet they are often simplified in applied analyses despite the possibility of nonlinear relationships with severe outcomes (6). These patterns are consistent with broader syntheses emphasizing that injury risk and severity are influenced by multiple interacting factors, including participant characteristics, equipment, and slope conditions (7).

Prevention in real-world resort settings requires identifying countermeasures that are feasible to implement at scale and then optimizing their deployment by understanding who benefits most and under what conditions. Helmet use has a strong evidence base for reducing head injury risk among skiers and snowboarders (8). However, other preventive practices beyond helmets, such as lower-extremity protection, warm-up behaviors, equipment checks (e.g., binding tests), and structured training or safety education, have been inadequately evaluated for severe outcomes, and the available evidence remains inconsistent. Complicating inference further, interventions and exposure may co-occur in ways that vary by risk profile. Moreover, interventions and exposure may co-occur in ways that vary by risk profile; for example, helmet wearers may exhibit higher patterns of risk-taking, motivating analytic approaches that move beyond average effects to examine effect heterogeneity (9).

Accordingly, we conducted a retrospective cross-sectional injury-severity study among injured skiers and snowboarders treated at resort medical clinics and emergency departments across three winter seasons, focusing on severe trauma likely to require admission and/or operative care. We aimed to: (i) evaluate independent associations of modifiable countermeasures and exposure-related variables with severe injury; (ii) characterize nonlinear dose–response relationships for key continuous factors; and (iii) test prespecified interaction effects to determine whether selected countermeasures confer stronger protection in higher-risk contexts. Finally, to support actionable safety planning, we translated key modifiable findings into scenario-standardized risk contrasts with uncertainty quantification. Because analyses are conditional on having sustained an injury, all contrasts reflect differences in severity among injured participants rather than injury incidence or population-level risk.

## 2 Materials and methods

### 2.1 Study design and setting

This retrospective cross-sectional injury-severity study was conducted over three consecutive winter seasons (2021–2024) at two major ski resorts in Zhangjiakou City, China. Injured patients were identified through resort medical clinic and emergency department records, and exposure- and behavior-related information was collected via post-injury questionnaire interviews. The study protocol was approved by the Ethics Committee for Sports Science Experiments of Beijing Sport University and was conducted in accordance with the Declaration of Helsinki. Written informed consent was obtained after clinical stabilization and prior to questionnaire administration. For enrolled adult participants who were temporarily unable to respond because of clinical condition, an authorized family member provided consent and completed the questionnaire on the participant’s behalf. Patients were not involved in the design, conduct, reporting, or dissemination of this research. In this study, recreational alpine skiing and snowboarding are collectively referred to as “snow sports.” The analytic focus was severe injury outcomes within an injured cohort; accordingly, the study was not designed to estimate injury occurrence risk in the general resort population.

### 2.2 Study population and data collection

Following established methodologies (10, 11), data collection and questionnaire-based interviews were conducted at on-site resort medical emergency centers between November 1 and March 31 of each winter season. Eligible injured patients were approached after clinical presentation, and interviews were completed within 48 h post-injury or, for severely injured participants, during hospitalization. For critically ill patients who were unable to respond, electronic questionnaires were administered to relatives after consent was obtained. A standardized, pre-designed questionnaire was administered to all participants. Of 2,756 eligible patients, 2,369 completed the questionnaire, yielding a response rate of 85.96%.

#### 2.2.1 Inclusion and exclusion criteria

Inclusion criteria were clinically confirmed trauma requiring medical attention, sustained during alpine skiing or snowboarding at the study resorts, in patients aged 18 years or older. Exclusion criteria comprised missing key treatment outcomes or core covariates, and refusal or inability to provide informed consent.

#### 2.2.2 Injury severity assessment

Information on initial management at resort medical clinics and EDs, together with subsequent in-hospital disposition, was collected for all participants. Injury severity was quantified using the Injury Severity Score (ISS), a widely used summary measure of overall trauma severity (12). Briefly, ISS is calculated as the sum of squares of the three highest Abbreviated Injury Scale (AIS) scores across the three most severely injured body regions; AIS is a standard scale for grading injury severity by body region (13). Consistent with trauma epidemiology standards, injuries were categorized as severe (ISS > 15) or non-severe (ISS ≤ 15) (14).

### 2.3 Study variables

#### 2.3.1 Demographic characteristics

Variables included sex, age, height, weight, body mass index (BMI) and sport discipline (alpine skiing/snowboarding).

#### 2.3.2 Physical status and medical history

Snow-sport injury history was defined as prior injuries incurred during alpine skiing or snowboarding (any injury involving the musculoskeletal system and/or internal organs) (15). Comorbidities were abstracted from in-hospital medical records and included a history of hypertension, respiratory diseases, and cardiovascular diseases.

#### 2.3.3 Snow-sport behavior and self-reported psychological characteristics

Snow-sport experience was defined as the duration from the first participation in alpine skiing or snowboarding to the current injury (16), categorized into four groups: first-time, ≤1 snow season, 2–4 snow seasons, and ≥5 snow seasons. Snow-sport skill level was classified into three grades (advanced, intermediate, and beginner) according to established criteria (17). Self-perceived speed was a subjective assessment of the participant’s own speed during snow-sport participation on the injury day (fast/slow) (18). Injury location was categorized as either within terrain parks or on ski slopes (19). Injury mechanisms were divided into four types: forward fall, backward fall, collision with another skier or snowboarder, and collision with obstacles (11). Emotional state during snow-sport participation was self-reported and classified into five categories: excited, nervous, scared, calm, and low-spirited. Warm-up before snow-sport participation was grouped into five categories: no warm-up, ≤10 min, 11–20 min, 21–30 min, and >30 min. Self-reported risk-taking tendency was categorized as more risk-taking or more cautious (20). Additional variables included: formal professional training experience (yes/no), physical health status on injury day (good/poor) and attention to safety education/alerts (yes/no).

#### 2.3.4 Equipment use

Use of protective equipment (helmet, snow goggles, hip pads, elbow pads, knee pads, and wrist guards) was recorded, along with history of binding testing (yes/no).

#### 2.3.5 Environmental conditions

The time and location of each incident were recorded through interviews and ski rescue team records. Meteorological data at the time of injury, including temperature (°C) and snow depth (cm), were obtained from the automated weather station nearest to the incident site. Stations were installed throughout the resorts at 50-m intervals and provided continuous minute-level monitoring. Weather conditions were classified as sunny, cloudy, or overcast based on relative humidity data from integrated temperature/humidity sensors; any recorded precipitation was classified as overcast. Snow quality was assessed using snow temperature and water content measured by a Snow Fork snow characteristic analyzer and a needle thermometer, and categorized as powder snow, icy snow, or wet snow (21).

### 2.4 Statistical analysis

Participant characteristics were summarized overall and stratified by injury severity. Continuous variables are presented as mean (standard deviation, SD), and categorical variables as n (%). Group comparisons were performed using *χ*^2^ tests or Fisher’s exact tests for categorical variables and Mann–Whitney *U* tests or independent *t*-tests for continuous variables, as appropriate. To mitigate small-sample bias and potential quasi-separation for the relatively uncommon outcome (prevalence approximately 14%), we fitted Firth-penalized logistic regression models to estimate odds ratios (ORs). The analysis modeled severe injury conditional on having sustained an injury, and thus does not estimate injury incidence in the general population.

*Hierarchical model building*. For reporting clarity, covariates were introduced in a revised three-stage framework. Model 1 included core modifiable factors (protective equipment, behavioral factors, experience-related factors, and training-related variables). Model 2 additionally adjusted for sex and restricted cubic spline (RCS; df = 4) terms for age and BMI. Model 3 (the primary fully adjusted model) further adjusted for physical condition, discipline, medical histories, weather/snow conditions (including RCS terms for temperature and snow depth), injury mechanism/location, and perceived speed and risk behavior. Continuous variables were modeled using RCS to allow nonlinearity, with overall Wald tests used to assess nonlinear components.

*Sensitivity analyses*. In the second step, robustness of Model 3 was assessed using: (a) standard logistic regression with heteroskedasticity-consistent (HC3) standard errors; (b) replacement of BMI (RCS, df = 4) with separate RCS (df = 4) for height and weight; and (c) reduction of knots for all continuous variables (age, BMI, temperature, snow depth) to df = 3 to assess spline specification stability.

*Effect modification analyses*. Prespecified multiplicative interaction blocks were evaluated in the fully adjusted model using joint Wald tests, with Benjamini-Hochberg false discovery rate control (*q* = 0.10). Significant interactions were visualized using marginal predicted probabilities. All predicted probabilities and contrasts are conditional on injury and should not be interpreted as population-level incidence estimates.

*Scenario-standardized impact metrics*. To translate modifiable findings into action-oriented metrics, counterfactual scenarios were generated in the fully adjusted model by shifting significant modifiable participant-level factors to their optimal levels (lowest marginal predicted risk). We estimated the population fraction preventable (PFP) standardized to the injured cohort, absolute risk differences (ARD), and the number needed to achieve the optimal level to avert one severe injury (NNTA). Uncertainty was quantified using 500 bootstrap resamples to obtain 95% confidence intervals. These standardized contrasts pertain to injured participants only and are not interpretable as population-level incidence reductions.

*Exploratory joint effects*. For selected exposure-intervention pairs, we assessed joint effects in separate fully adjusted models that included interaction terms, reporting odds ratios for combined categories relative to high-exposure/no-intervention references. Given anticipated sparse strata and the relatively rare outcome, these analyses were considered exploratory, with emphasis placed on estimate stability rather than formal inference.

*A complete-case analysis was performed*. All tests were two-sided, and *p* value < 0.05 indicated statistical significance unless otherwise specified (interaction screening controlled by *q* = 0.10). All analyses were performed in Python 3.13.5 (Anaconda distribution; Python Software Foundation), primarily using the pandas, numpy, statsmodels, patsy, scikit-learn, and matplotlib packages. Firth-penalized logistic regression was implemented using a custom penalized-likelihood iterative fitting routine with a maximum of 200 iterations and a convergence criterion defined as a maximum absolute parameter step <1 × 10^−8^.

## 3 Results

### 3.1 Baseline characteristics of study population

A total of 2,369 injured participants (skiers and snowboarders) were included, of whom 339 (14.3%) sustained severe injuries and 2,030 (85.7%) had non-severe injuries. As shown in Table 1, several modifiable factors differed by injury severity. Risk-taking behavior was more common among severe cases (32.8% vs. 11.9% in cautious participants; *p* < 0.001). Longer snow-sport experience and beginner skill level were also associated with a higher proportion of severe injury (≥5 seasons: 22.7%; beginners: 33.3% vs. advanced: 9.8%; both *p* < 0.001). Non-use of knee protectors was associated with a higher proportion of severe injury (20.3% vs. 13.6% in users; *p* = 0.006). Warm-up duration differed across severity groups, with the highest proportion of severe injury observed among participants reporting warm-up >30 min (34.3% vs. 6.0% with no warm-up; *p* < 0.001). Severe cases more frequently reported fast self-perceived speed (17.6% vs. 11.6%; *p* < 0.001) and alpine skiing rather than snowboarding (17.4% vs. 12.0%; *p* < 0.001). Injuries occurring on regular slopes (vs. terrain parks) were more common among severe cases (15.6% vs. 9.4%; *p* < 0.001). Males had slightly higher severe injury rates than females (16.1% vs. 12.8%; *p* = 0.025). Environmental and medical factors associated with severe injury included lower snow depth (15.3 cm vs. 18.1 cm; *p* < 0.001), warmer temperatures (−10.5 °C vs. − 11.7 °C; *p* = 0.022), and histories of musculoskeletal disorders (*p* = 0.007) or hypertension (*p* = 0.008).

**Table 1:** Characteristics of snow-sport participants with severe and non-severe injuries.

Characteristic	Level	Non-severe injury (*n* = 2,030)	Severe injury (*n* = 339)	Severe injury within level (%)	*p* value
Continuous variables					
Age, years		29.4 (6.4)	29.7 (5.4)		0.349
Height, cm		169.0 (7.5)	169.9 (6.9)		0.033
Weight, kg		60.3 (10.2)	60.9 (9.7)		0.292
BMI, kg/m^2^		21.0 (2.4)	21.0 (2.1)		0.997
Temperature, °C		−11.7 (8.3)	−10.5 (8.7)		0.022
Snow depth, cm		18.1 (9.6)	15.3 (9.7)		<0.001
Categorical variables					
Sex	Male	900 (83.9)	173 (16.1)	16.1	0.025
	Female	1,130 (87.2)	166 (12.8)	12.8	
Discipline	Alpine skiing	835 (82.6)	176 (17.4)	17.4	<0.001
	Snowboarding	1,195 (88.0)	163 (12.0)	12.0	
Physical condition	Good	1,998 (85.6)	337 (14.4)	14.4	0.243
	Poor	32 (94.1)	2 (5.9)	5.9	
Risk behavior	More risk-taking	180 (67.2)	88 (32.8)	32.8	<0.001
	More cautious	1,850 (88.1)	251 (11.9)	11.9	
Self-perceived speed	Fast	877 (82.4)	187 (17.6)	17.6	<0.001
	Slow	1,153 (88.4)	152 (11.6)	11.6	
Injury location	On ski slopes	1,575 (84.4)	292 (15.6)	15.6	<0.001
	Terrain parks	455 (90.6)	47 (9.4)	9.4	
Injury mechanism	Forward fall	558 (88.4)	73 (11.6)	11.6	0.001
	Backward fall	553 (87.9)	76 (12.1)	12.1	
	Collision with other participants	673 (81.9)	149 (18.1)	18.1	
	Collision with obstacles	246 (85.7)	41 (14.3)	14.3	
Snow quality	Powder snow	1,838 (85.4)	313 (14.6)	14.6	0.505
	Icy snow	163 (88.6)	21 (11.4)	11.4	
	Wet snow	29 (85.3)	5 (14.7)	14.7	
Weather condition	Sunny	943 (84.5)	173 (15.5)	15.5	0.166
	Cloudy	810 (87.4)	117 (12.6)	12.6	
	Overcast	277 (85.0)	49 (15.0)	15.0	
Knee protector use	No	196 (79.7)	50 (20.3)	20.3	0.006
	Yes	1,834 (86.4)	289 (13.6)	13.6	
Helmet use	No	90 (86.5)	14 (13.5)	13.5	0.913
	Yes	1,940 (85.7)	325 (14.3)	14.3	
Goggle use	No	365 (87.7)	51 (12.3)	12.3	0.216
	Yes	1,665 (85.3)	288 (14.7)	14.7	
Hip protector use	No	1,001 (88.0)	136 (12.0)	12.0	0.002
	Yes	1,029 (83.5)	203 (16.5)	16.5	
Elbow protector use	No	451 (88.3)	60 (11.7)	11.7	0.072
	Yes	1,579 (85.0)	279 (15.0)	15.0	
Wrist protector use	No	806 (88.1)	109 (11.9)	11.9	0.010
	Yes	1,224 (84.2)	230 (15.8)	15.8	
Binding release test	No	481 (91.8)	43 (8.2)	8.2	<0.001
	Yes	1,549 (84.0)	296 (16.0)	16.0	
Professional training	No	1,072 (90.6)	111 (9.4)	9.4	<0.001
	Yes	958 (80.8)	228 (19.2)	19.2	
Safety education	No	140 (89.7)	16 (10.3)	10.3	0.168
	Yes	1,890 (85.4)	323 (14.6)	14.6	
Mood state	Calm	231 (82.8)	48 (17.2)	17.2	0.338
	Excited	593 (86.8)	90 (13.2)	13.2	
	Nervous	681 (86.8)	104 (13.2)	13.2	
	Scared	414 (84.0)	79 (16.0)	16.0	
	Low-spirited	111 (86.0)	18 (14.0)	14.0	
Warm-up duration	No warm-up	110 (94.0)	7 (6.0)	6.0	<0.001
	≤10 min	765 (87.9)	105 (12.1)	12.1	
	11–20 min	825 (84.1)	156 (15.9)	15.9	
	21–30 min	284 (85.8)	47 (14.2)	14.2	
	>30 min	46 (65.7)	24 (34.3)	34.3	
Skiing experience	First-time	303 (87.1)	45 (12.9)	12.9	<0.001
	≤1 snow season	517 (90.4)	55 (9.6)	9.6	
	2–4 snow seasons	931 (85.6)	157 (14.4)	14.4	
	≥5 snow seasons	279 (77.3)	82 (22.7)	22.7	
Self-reported skill level	Beginner	44 (66.7)	22 (33.3)	33.3	<0.001
	Intermediate	675 (79.5)	174 (20.5)	20.5	
	Advanced	1,311 (90.2)	143 (9.8)	9.8	
Musculoskeletal disease history	No	1,684 (86.6)	260 (13.4)	13.4	0.007
	Yes	346 (81.4)	79 (18.6)	18.6	
Hypertension history	No	1,786 (86.4)	280 (13.6)	13.6	0.008
	Yes	244 (80.5)	59 (19.5)	19.5	
Cardiovascular disease history	No	2,027 (85.7)	338 (14.3)	14.3	1.000
	Yes	3 (75.0)	1 (25.0)	25.0	
Respiratory disease history	No	1,939 (85.7)	324 (14.3)	14.3	1.000
	Yes	91 (85.8)	15 (14.2)	14.2	

BMI, body mass index. Continuous variables are presented as mean (SD). Categorical variables are presented as *n* (%). Percentages in the “Non-severe injury” and “Severe injury” columns are row percentages within each variable level; the “Severe injury within level (%)” column is provided to enhance clinical interpretability. *p* values were obtained using Welch’s *t*-test for continuous variables and Pearson’s chi-square test for categorical variables.

### 3.2 Independent associations of modifiable factors and risk exposures with severe snow-sport injury

Multivariable Firth-penalized logistic regression results are summarized in Table 2. In the minimally adjusted model (Model 1; core modifiable factors), knee protector use (OR = 0.48, 95% CI: 0.33–0.70; *p* < 0.001) and advanced skill level (OR = 0.35, 95% CI: 0.19–0.66; *p* = 0.001) were associated with lower odds of severe injury, whereas professional training was associated with higher odds (OR = 1.50, 95% CI: 1.13–2.00; *p* = 0.005). These associations remained robust after adding sex, age and BMI (Model 2). In the fully adjusted model (Model 3), knee protector use (OR = 0.57, 95% CI: 0.39–0.84; *p* = 0.005), advanced skill level (OR = 0.46, 95% CI: 0.23–0.90; *p* = 0.023), more cautious risk behavior (OR = 0.46, 95% CI: 0.33–0.65; *p* < 0.001), and snowboarding rather than alpine skiing (OR = 0.67, 95% CI: 0.51–0.88; *p* = 0.005) were associated with lower odds of severe injury, whereas collision with other participants (vs. forward fall) was associated with higher odds (OR = 1.51, 95% CI: 1.10–2.08; *p* = 0.011). Sensitivity analyses demonstrated high robustness across model specifications (Supplementary Table S1), with key associations (knee protector use, cautious behavior, advanced skill level, and snowboarding) remaining consistent in direction, magnitude, and statistical significance.

**Table 2:** Multivariable firth-penalized logistic regression models for severe snow-sport injury.

Predictor	Model 1 OR (95% CI)	*p*	Model 2 OR (95% CI)	*p*	Model 3 OR (95% CI)	*p*
Core modifiable factors: equipment and training characteristics						
Knee protector use (yes vs. no)	0.48 (0.33–0.70)	<0.001^***^	0.50 (0.34–0.72)	<0.001^***^	0.57 (0.39–0.84)	0.005^**^
Helmet use (yes vs. no)	0.95 (0.52–1.72)	0.856	0.99 (0.54–1.83)	0.984	0.87 (0.47–1.62)	0.654
Snow goggle use (yes vs. no)	0.84 (0.59–1.18)	0.308	0.85 (0.60–1.21)	0.363	0.78 (0.55–1.11)	0.172
Hip protector use (yes vs. no)	1.15 (0.89–1.48)	0.295	1.17 (0.90–1.52)	0.243	1.13 (0.86–1.47)	0.385
Elbow protector use (yes vs. no)	1.21 (0.87–1.68)	0.257	1.21 (0.86–1.68)	0.271	1.18 (0.84–1.66)	0.326
Wrist protector use (yes vs. no)	1.19 (0.89–1.58)	0.233	1.18 (0.88–1.57)	0.264	1.21 (0.90–1.62)	0.205
Binding test history (yes vs. no)	1.47 (1.00–2.16)	0.050	1.45 (0.98–2.15)	0.062	1.35 (0.91–2.01)	0.134
Professional training (yes vs. no)	1.50 (1.13–2.00)	0.005^**^	1.49 (1.12–1.99)	0.007^**^	1.34 (1.00–1.79)	0.053
Core modifiable factors: behavioral and psychological characteristics						
Attention to safety education/alerts (yes vs. no)	0.85 (0.47–1.51)	0.571	0.86 (0.47–1.57)	0.630	0.93 (0.51–1.71)	0.826
Emotional state: excited vs. calm	0.90 (0.60–1.34)	0.595	0.94 (0.63–1.41)	0.774	1.10 (0.72–1.68)	0.654
Emotional state: nervous vs. calm	1.07 (0.72–1.59)	0.738	1.11 (0.74–1.66)	0.606	1.20 (0.79–1.82)	0.394
Emotional state: scared vs. calm	1.16 (0.76–1.75)	0.493	1.19 (0.78–1.81)	0.420	1.12 (0.73–1.74)	0.603
Emotional state: low-spirited vs. calm	0.91 (0.50–1.68)	0.767	0.92 (0.49–1.71)	0.786	0.95 (0.50–1.81)	0.887
Warm-up: ≤10 min vs. none	1.52 (0.69–3.33)	0.299	1.52 (0.67–3.47)	0.315	1.62 (0.71–3.68)	0.251
Warm-up: 11–20 min vs. none	1.47 (0.66–3.27)	0.343	1.41 (0.61–3.26)	0.418	1.46 (0.63–3.36)	0.378
Warm-up: 21–30 min vs. none	0.97 (0.41–2.29)	0.948	0.91 (0.37–2.23)	0.837	0.96 (0.39–2.34)	0.922
Warm-up: >30 min vs. none	2.67 (1.02–7.00)	0.045^*^	2.60 (0.96–7.05)	0.061	2.58 (0.94–7.09)	0.067
Experience: ≤1 snow season vs. first-time	0.82 (0.53–1.26)	0.363	0.83 (0.53–1.29)	0.408	1.06 (0.67–1.69)	0.805
Experience: 2–4 snow seasons vs. first-time	0.96 (0.66–1.40)	0.846	0.94 (0.64–1.38)	0.766	1.19 (0.79–1.78)	0.408
Experience: ≥5 snow seasons vs. first-time	1.24 (0.80–1.91)	0.339	1.22 (0.78–1.91)	0.377	1.32 (0.83–2.10)	0.242
Skill level: intermediate vs. beginner	0.59 (0.33–1.05)	0.073	0.58 (0.32–1.05)	0.071	0.65 (0.35–1.20)	0.169
Skill level: advanced vs. beginner	0.35 (0.19–0.66)	0.001^**^	0.35 (0.19–0.67)	0.002^**^	0.46 (0.23–0.90)	0.023^*^
Demographic variables						
Sex: female vs. male	–	–	0.88 (0.65–1.20)	0.417	0.88 (0.64–1.20)	0.414
Age: per year (RCS)^†^	–	–	–	–	–	–
BMI: per kg/m^2^ (RCS)^†^	–	–	–	–	–	–
Clinical, environmental and incident-related covariates						
Poor physical condition vs. good	–	–	–	–	0.31 (0.07–1.41)	0.130
Discipline: snowboarding vs. alpine skiing	–	–	–	–	0.67 (0.51–0.88)	0.005^**^
Musculoskeletal history (yes vs. no)	–	–	–	–	1.32 (0.98–1.78)	0.072
Hypertension history (yes vs. no)	–	–	–	–	1.20 (0.84–1.72)	0.313
Cardiovascular history (yes vs. no)	–	–	–	–	0.97 (0.08–11.69)	0.979
Respiratory disease history (yes vs. no)	–	–	–	–	1.22 (0.67–2.20)	0.515
Snow condition: icy vs. powder	–	–	–	–	0.70 (0.35–1.41)	0.320
Snow condition: wet vs. powder	–	–	–	–	0.42 (0.14–1.33)	0.141
Weather: cloudy vs. sunny	–	–	–	–	0.77 (0.56–1.06)	0.109
Weather: overcast vs. sunny	–	–	–	–	1.46 (0.85–2.49)	0.169
Injury mechanism: backward fall vs. forward fall	–	–	–	–	1.08 (0.76–1.54)	0.674
Injury mechanism: collision with other participants vs. forward fall	–	–	–	–	1.51 (1.10–2.08)	0.011^**^
Injury mechanism: collision with obstacles vs. forward fall	–	–	–	–	1.18 (0.76–1.81)	0.462
Injury location: terrain parks vs. ski slopes	–	–	–	–	0.81 (0.57–1.15)	0.250
Self-perceived speed: slow vs. fast	–	–	–	–	0.90 (0.69–1.18)	0.453
Risk behavior: more cautious vs. more risk-taking	–	–	–	–	0.46 (0.33–0.65)	<0.001^***^
Temperature: per °C (RCS)^†^	–	–	–	–	–	–
Snow depth: per cm (RCS)^†^	–	–	–	–	–	–

Data are presented as odds ratio (OR) with 95% confidence interval (CI). BMI, body mass index. RCS, restricted cubic splines. Firth’s penalized likelihood logistic regression was used to reduce small-sample bias and potential separation. Model 1 included core modifiable factors. Model 2 additionally adjusted for sex and RCS terms for age and BMI. Model 3 further adjusted for physical condition, discipline, medical histories, weather/snow conditions, injury mechanism/location, and perceived speed and risk behavior. ^†^Age, BMI, temperature, and snow depth were modeled with RCS and are therefore not displayed as single-effect ORs. *p < 0.05; **p < 0.01; ***p < 0.001.

### 3.3 Non-linear dose–response relationships

**Figure 1:**
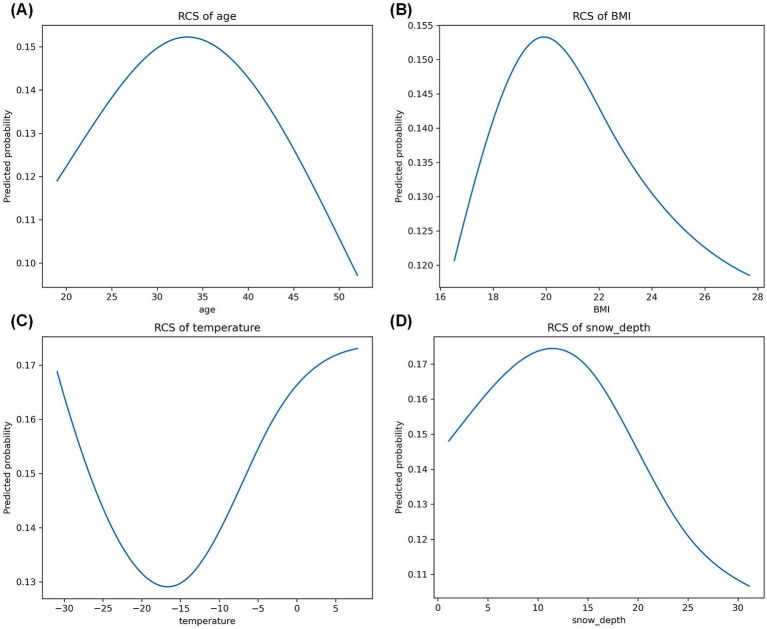
Nonlinear associations of continuous predictors with severe snow-sport injury using restricted cubic splines. RCS, restricted cubic splines. BMI, body mass index. **(A)** RCS of age. **(B)** RCS of BMI. **(C)** RCS of temperature. **(D)** RCS of snow depth.

In the fully adjusted model, RCS analyses suggested potentially non-linear patterns for several continuous variables, whereas the overall Wald tests indicated statistical evidence only for snow depth (Supplementary Table S2; Figure 1). Age (Figure 1A) and BMI (Figure 1B) showed visually inverted U-shaped patterns, with peak predicted probabilities of severe injury at approximately 32–35 years (predicted probability approximately 0.15) and 19–20 kg/m^2^ (predicted probability approximately 0.152), respectively, followed by declines toward the extremes. Temperature (Figure 1C) showed a visually U-shaped pattern, with the lowest predicted probability at approximately −15 to −20 °C (predicted probability approximately 0.13) and increasing risk toward warmer conditions (plateau approximately 0.16–0.17 near 0–5 °C). Snow depth (Figure 1D) also displayed an inverted U-shaped pattern, with the highest predicted probability at shallow depths (8–12 cm; approximately 0.165–0.17) and a marked reduction at greater depths (20–30 cm; approximately 0.11). However, among the spline-modeled continuous variables, only snow depth showed a statistically significant overall association in the fully adjusted model (overall Wald *p* = 0.010), whereas age, BMI, and temperature did not (all *p* > 0.05). Sensitivity analyses replacing BMI with separate RCS terms for height and weight yielded comparatively flat relationships (predicted probabilities approximately 0.143 across ranges; overall non-linearity *p* > 0.05; Supplementary Figure S1), supporting the primary specification using BMI.

### 3.4 Multiplicative interaction effects between modifiable factors and risk exposures

**Figure 2:**
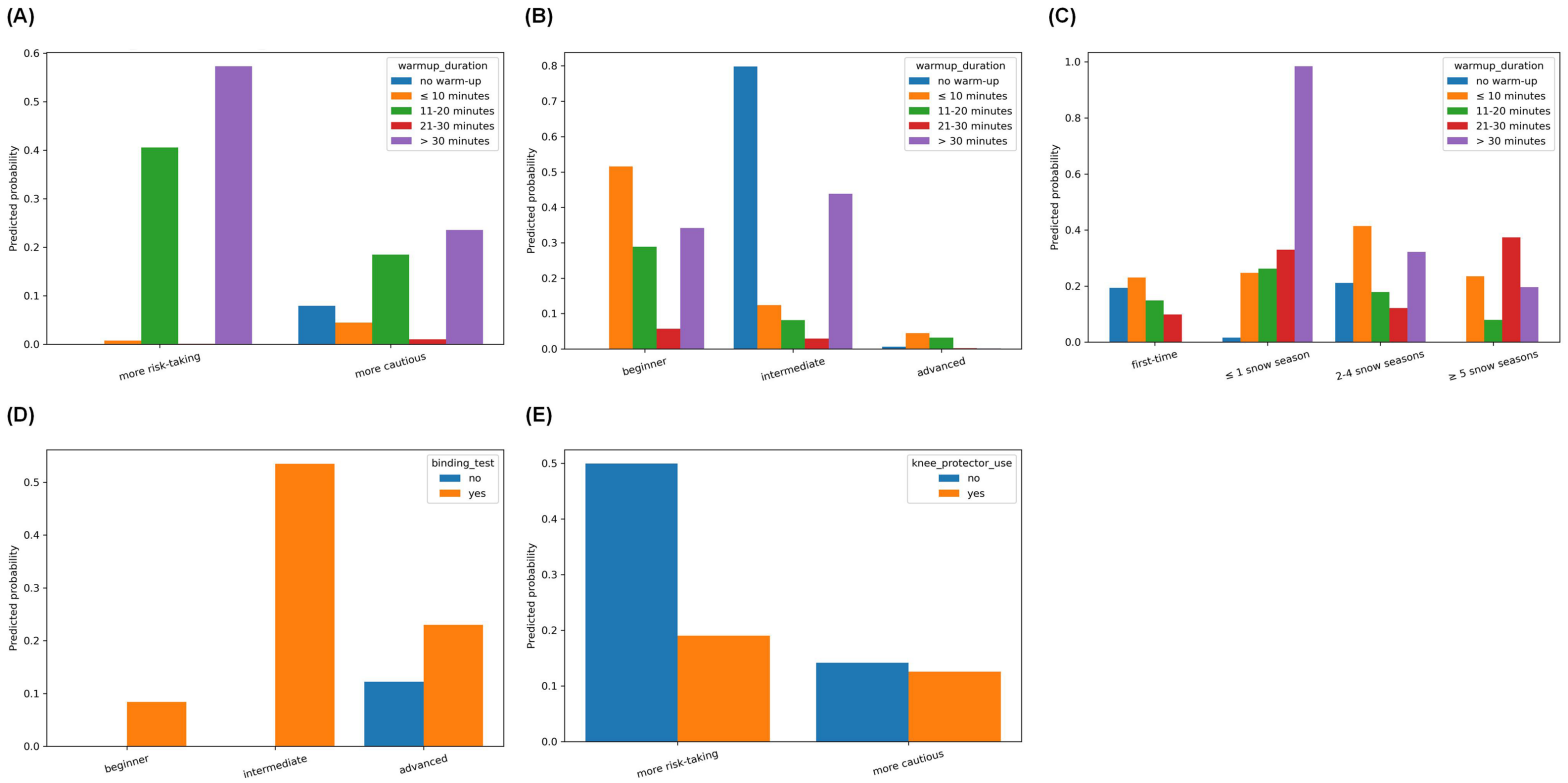
Significant multiplicative interaction effects on severe snow-sport injury risk. **(A)** Interaction between risk-taking behavior and warm-up duration. **(B)** Interaction between skill level and warm-up duration. **(C)** Interaction between snow-sport experience and warm-up duration. **(D)** Interaction between skill level and binding test. **(E)** Interaction between risk-taking behavior and knee protector use.

Multiplicative interaction testing in the fully adjusted model identified five significant effects after Benjamini-Hochberg false discovery rate correction (*q* = 0.10; Supplementary Table S3). Warm-up duration significantly modified associations with snow-sport experience, risk-taking behavior, and skill level (joint Wald *p* < 0.001 for each). Binding test modified the association with skill level (*p* < 0.001), and knee protector use modified the association with risk-taking behavior (*p* = 0.001). Marginal predicted probabilities (Figure 2) suggested stronger protective contrasts for interventions within higher-risk subgroups. Longer warm-up durations (>30 min) were associated with substantially lower predicted probabilities of severe injury among participants with low experience, beginner skill level, or risk-taking behavior, with smaller contrasts among experienced, advanced, or cautious participants. Binding tests (Figure 2D) and knee protector use (Figure 2E) similarly showed larger protective contrasts among beginners and risk-takers, respectively.

### 3.5 Potential preventive impact of optimizing modifiable factors

To aid practical interpretation, we estimated scenario-standardized contrasts for selected modifiable factors in the fully adjusted Firth-penalized logistic regression model (Table 3). These metrics summarize modeled differences in severe-injury probability within the injured cohort under alternative factor levels and are presented as the PFP, ARD, and NNTA. They should be interpreted as planning-oriented summaries rather than direct causal effect estimates. Optimizing warm-up duration to 21–30 min (vs. no warm-up) produced the largest point estimate (PFP = 27.7, 95% CI: −156.8 to 87.2%), corresponding to an ARD of 3.96 percentage points (95% CI: −14.94 to 17.49) and an NNTA of 25. Achieving advanced skill level (vs. beginner) was associated with a PFP of 14.4% (95% CI: −46.0 to 54.8%; ARD = 2.07 percentage points, 95% CI: −5.10 to 9.58; NNTA = 48), although feasibility is constrained by training requirements. Universal knee protector use yielded a smaller point estimate (PFP = 4.9, 95% CI: −11.6 to 23.5%; ARD = 0.70 percentage points, 95% CI: −1.23 to 2.53; NNTA = 142). Confidence intervals were wide and included the null, indicating substantial uncertainty. These estimates should be interpreted as scenario-standardized projections within the injured cohort rather than population-level incidence reductions (Figure S2).

**Table 3:** Scenario-standardized prevention metrics for selected modifiable factors associated with severe snow-sport injury among injured participants.

Variable	Optimal level	PFP (%) (95% CI)	ARD (percentage points) (95% CI)	NNTA	Clinical feasibility	Public health priority
Knee protector use	Yes	4.9% (−11.6 to 23.5%)	0.70 (−1.23 to 2.53)	142	High (simple adoption)	Moderate
Warm-up duration	21–30 min (vs no warm-up)	27.7% (−156.8 to 87.2%)	3.96 (−14.94 to 17.49)	25	High	High (if confirmed)
Skier level	Advanced (vs beginner)	14.4% (−46.0 to 54.8%)	2.07 (−5.10 to 9.58)	48	Low (requires long-term training)	Low

CI, confidence interval; PFP, population fraction preventable; ARD, absolute risk difference; NNTA, number needed to achieve the optimal level to avert one severe injury. PFP was calculated as (baseline predicted risk − counterfactual predicted risk under the optimal level)/baseline predicted risk, with risks averaged over the analytic population of injured participants. The baseline predicted probability of severe injury in the injured cohort was 14.3%. ARD is expressed in percentage points, with positive values indicating risk reduction. NNTA was calculated conditional on injury and rounded to the nearest integer. Bootstrap 95% CIs were obtained from 500 replications. Wide confidence intervals reflect substantial uncertainty related to sparse data and the relatively uncommon outcome. These estimates are scenario-standardized, model-based planning-oriented contrasts within the injured cohort and should not be interpreted as direct causal effect estimates or population-level incidence reductions.

### 3.6 Exploratory combined effects of selected exposure-intervention pairs

Exploratory joint effects for pre-specified exposure-intervention pairs (perceived speed or risk-taking behavior with knee protector use, helmet use, professional training, safety education, or warm-up duration) in fully adjusted models produced highly variable point estimates with extremely wide confidence intervals due to sparse strata (Supplementary Table S4). No significant synergistic or buffering effects were detected, and these results did not provide reliable evidence beyond the main-effect and interaction findings.

## 4 Discussion

This injury-severity study among injured recreational skiers and snowboarders suggests that severe outcomes are associated with a combination of modifiable practices, behavioral/skill-related factors, injury mechanisms, and environmental conditions. In fully adjusted analyses, knee protector use, cautious risk behavior, and advanced skill level were associated with lower odds of severe injury, whereas collisions with other participants were associated with higher severity. Beyond average associations, we identified nonlinear dose–response patterns for age, BMI, temperature, and snow depth, and observed effect modification indicating that warm-up duration and binding tests may confer larger protective contrasts in higher-risk subgroups (e.g., beginners and self-reported risk-takers). Scenario-standardized metrics translated selected modifiable factors into prevention-oriented contrasts, although estimates were imprecise and should be interpreted cautiously.

Our findings align with the broader snow-sport injury literature emphasizing the roles of participant ability, behavior, and context (7, 22), while extending prior work by focusing on severe versus non-severe outcomes among individuals who were already injured. This severity-conditional perspective is clinically relevant for admission and resource use but differs conceptually from incidence studies that employ exposure denominators (e.g., skier-days). Conditioning on injury may induce selection (collider) mechanisms, which can attenuate or alter associations relative to exposure-denominator designs, particularly for variables strongly related to injury occurrence (23). Accordingly, our results should be interpreted as determinants of severity among injured participants rather than determinants of injury risk in the resort population.

The association between collisions and higher odds of severe injury reinforces person-to-person impacts as a high-energy mechanism and supports the need for upstream, resort-level safety measures in addition to individual counseling. Collisions plausibly involve high relative velocity and limited time for protective responses, increasing the likelihood of serious trauma. Registry evidence from Austria similarly highlights clinically important injury patterns in collisions (3). From an operational perspective, this pattern supports layered resort strategies such as crowding management, clearer signage at merge zones, and targeted messaging in high-traffic corridors. Behavioral risk regulation and skill level are also plausible levers for reducing severe outcomes by lowering impact energy and preventing loss-of-control events.

In addition to these mechanisms, our baseline comparisons indicated that severe injuries were more frequent among participants reporting longer snow-sport experience (≥5 seasons) and among beginners compared with advanced participants. The strong gradient by skill level is consistent with reduced control margins and less effective protective responses during loss-of-balance events, which may increase the likelihood that an incident escalates to severe trauma. The pattern observed for longer experience may appear counterintuitive if interpreted as a population-level risk relationship; however, in an injury-severity analysis conditional on having sustained an injury, experienced participants who present for medical care may represent a subgroup exposed to higher-intensity conditions (e.g., higher speed, more challenging terrain selection, or greater collision exposure) that elevate the probability of severe outcomes once an injury occurs. Clinical profiles of winter sports injuries also vary by participant characteristics and exposure context, as described in prospective clinical observations (24). Importantly, because our analyses are conditional on injury, these associations should be interpreted as determinants of severity among injured patients rather than determinants of injury incidence in the overall slope population, and selection (collider) mechanisms may contribute to observed contrasts.

Prior field research documents substantial variability in speed shaped by individual and contextual factors (4). This provides a mechanistic bridge whereby risk-taking and fast speed increase kinetic energy and reduce margins for correction, making severe outcomes more likely once an incident occurs. The protective association observed for knee protector use is promising but should be interpreted cautiously given limited snow-sport-specific efficacy evidence and potential correlation with safety orientation. Evidence for some protective equipment in snow sports (e.g., helmets; wrist guards) is more established (7, 8, 25), whereas knee protectors warrant further evaluation using injury-type-specific outcomes and designs with improved exposure measurement.

A key contribution of this study is the use of flexible spline modeling to examine whether associations for age, BMI, temperature, and snow depth departed from simple linearity, thereby allowing the exploration of possible “risk windows” rather than assuming uniform gradients. In the fully adjusted model, these spline analyses revealed visually suggestive patterns across several variables, although the overall Wald tests indicated statistically robust evidence only for snow depth. RCS methods are recommended when linearity is uncertain because categorization can obscure thresholds and distort functional form (26). Practically, the observed patterns imply that condition-aware messaging and operational decisions may be more effective when focused on specific ranges of environmental conditions where severity probability is highest, rather than assuming monotonic changes. Our findings complement resort-based evidence that injury patterns vary with weather and snow conditions (21), while adding nuance by focusing on severity among injured participants and allowing flexible functional forms.

The most prevention-relevant and novel finding is the evidence of effect modification for warm-up duration, indicating that protective contrasts may be larger among higher-risk participants (e.g., novices and risk-takers). Warm-up has been proposed as an injury-prevention strategy across sports, with systematic evidence supporting injury reductions for structured exercise-based prevention programs, although effects vary by sport and program design (27, 28). In our analysis, marginal predictions suggested larger absolute differences across warm-up categories in higher-risk strata, consistent with mechanisms such as neuromuscular activation, proprioceptive priming, and improved early-run coordination that may reduce loss-of-control events escalating into severe trauma. From an implementation standpoint, warm-up promotion is low-cost and scalable; our results support prioritizing warm-up messaging for higher-risk participants (e.g., at ski school intake or rental-shop encounters), while coupling it with skill-focused instruction and collision-risk mitigation.

Counterfactual-style summary metrics can help translate regression findings into more interpretable planning contrasts, but they should not be understood as defining the only actionable elements of prevention. In our study, scenario-standardized metrics suggested that warm-up optimization could yield the largest preventable fraction within the injured cohort, but these projections should be interpreted as planning tools rather than causal guarantees. Model-based preventable fractions derived from logistic models depend on modeling assumptions and can be unstable under sparse data, particularly for interaction-sensitive contrasts (29, 30). In our study, uncertainty was substantial, underscoring that the estimated prevention impact should be viewed as a comparative scenario exercise and a rationale for pragmatic evaluation rather than definitive effect sizes. Table 3 therefore summarizes selected modifiable factors for which scenario-standardized contrasts could be estimated directly from the fully adjusted model, rather than serving as a complete inventory of all prevention-relevant findings. Environmental conditions, collision-related mechanisms, and other contextual factors remain highly important for clinicians, coaches, resort managers, policymakers, and researchers, even when they are not expressed in the same metric format. From a broader systems perspective, participant-level practices and resort-level operational factors should be interpreted as complementary components of severe-injury prevention. In this regard, weather, temperature, snow depth, and snow quality can still inform condition-aware patrol deployment, warning systems, slope management, grooming schedules, traffic control, and targeted safety communication during higher-risk condition windows.

Taken together, these findings support a safety-management approach that prioritizes severe-outcome mitigation through targeted, layered interventions. First, target severe-injury prevention where vulnerability is greatest. Warm-up promotion and skills-focused messaging may yield the largest practical gains when prioritized for higher-risk subgroups (e.g., novices and self-identified risk-takers), rather than delivered as uniform “one-size-fits-all” advice. Second, treat collision risk as a system-level hazard. Resort-level controls (traffic management, intersection/merge-zone design, visibility improvements, crowding management, and speed-differentiated slope policies) should be central components of severe-injury mitigation, complementing individual behavior change. Third, use condition-aware safety operations. Non-linear patterns for temperature and snow depth suggest that safety communication and patrol resources may be most efficiently intensified during specific “high-risk condition windows,” rather than assuming constant incremental risk changes. Fourth, layer countermeasures rather than relying on equipment alone. Protective equipment and equipment checks (e.g., binding tests) should be implemented as part of a layered strategy that also includes behavioral preparation and risk communication, acknowledging potential behavioral adaptation. Finally, design surveillance around severe endpoints. Routine resort injury surveillance can track severe outcomes using standardized trauma definitions (e.g., ISS > 15) to inform targeted interventions and evaluate safety policy changes over time.

Strengths include the focus on a severe endpoint, a large resort/ED sample across three seasons, and analytic strategies tailored to a relatively uncommon outcome and potential nonlinearity. Firth-penalized models reduce small-sample bias and instability under quasi-separation, and RCS provide flexible estimation of dose–response patterns (23, 26, 31). Presenting effect modification using marginal predicted probabilities improves interpretability compared with interaction terms alone. Several limitations should be noted. First, the retrospective cross-sectional design and self-reported measures introduce potential residual confounding and misclassification; for example, warm-up duration may correlate with unmeasured factors such as fitness, instruction quality, fatigue, or anticipated risk. Several behavioral variables, particularly emotional state and risk-taking tendency, were self-reported and may be subject to measurement error. This limitation is more salient for proxy-completed questionnaires, for which internal states may be less reliably captured than directly observable exposures. Second, because analyses were conditional on injury, findings may not generalize to incidence in the overall slope population and may be influenced by selection/collider mechanisms (23). Third, the restriction to adults limits generalizability to children and adolescents, who represent an important snow-sport population with potentially different injury patterns and supervision contexts. Future studies are needed to specifically investigate the risk profiles and severity determinants of snow-sport injuries in children and adolescents aged under 18 years. Fourth, sparse joint strata led to imprecision for interaction and scenario contrasts despite penalization, so interaction results should be interpreted as evidence of heterogeneity patterns rather than precise subgroup effects. Finally, several resort-level factors (e.g., crowding, surface hardness, visibility, enforcement intensity) were not directly measured and may contribute to both exposure patterns and severity.

## 5 Conclusion

In conclusion, among injured adult skiers and snowboarders, severe outcomes reflect an interplay of exposure intensity, injury mechanisms, environmental conditions, and modifiable preventive practices. The combination of non-linear risk windows and subgroup-specific benefits strongly argues for the necessity of adopting a risk-stratified safety strategy: prioritize warm-up promotion and skill-focused messaging for novices and risk-takers, encourage scalable protective practices (e.g., knee protectors and binding checks where appropriate), and pair these with resort-level measures to reduce collision hazards. Prospective studies integrating exposure denominators and implementation evaluation are needed to determine whether targeted warm-up promotion and related countermeasures causally reduce severe injury burden and to quantify their effectiveness in real-world resort settings.

## Data Availability

The raw data supporting the conclusions of this article will be made available by the authors, without undue reservation.
