# Supplementary material for: Associated factors, nonlinear risk patterns and effect heterogeneity of severe injuries in recreational skiers and snowboarders
Source: Front Public Health. 2026 Apr 13;14:1814705. doi: 10.3389/fpubh.2026.1814705 (PMC13111118; doi:10.3389/fpubh.2026.1814705)
Supplement: Supplementary file 1 [file Data_sheet_1.docx]

**TABLE S1 Sensitivity analyses comparing alternative model specifications for severe snow-sport injury.**

| **Variable Group** | **Variable** | **Level** | **Sensitivity 1 (Standard logit+HC3)**  **OR (95% CI)**  **P value** | **Sensitivity 2 (Height/Weight)**  **OR (95% CI)**  **P value** | **Sensitivity 3**  **(RCS df=3)**  **OR (95% CI)**  **P value** |
| --- | --- | --- | --- | --- | --- |
| **1. Core Modifiable Factors** | **Protective Equipment** | Knee protector use (Yes) | **0.57(0.39–0.85) 0.005**** | **0.60 (0.40–0.89) 0.012*** | **0.57 (0.39–0.84) 0.005**** |
|  |  | Helmet use (Yes) | 0.87 (0.46–1.63) 0.656 | 0.85 (0.45–1.59) 0.610 | 0.89 (0.48–1.67) 0.720 |
|  |  | Wrist protector use (Yes) | 1.21 (0.90–1.63) 0.210 | 1.21 (0.90–1.63) 0.217 | 1.21 (0.90–1.62) 0.217 |
|  |  | Hip protector use (Yes) | 1.13 (0.86–1.48) 0.390 | 1.14 (0.87–1.50) 0.343 | 1.11 (0.85–1.46) 0.431 |
|  |  | Elbow protector use (Yes) | 1.18 (0.84–1.67) 0.332 | 1.17 (0.83–1.64) 0.380 | 1.19 (0.84–1.67) 0.324 |
|  |  | Goggle use (Yes) | 0.78 (0.55–1.12) 0.178 | 0.79 (0.55–1.14) 0.209 | 0.78 (0.54–1.12) 0.173 |
|  | **Behavioral Factors** | Risk behavior (More cautious) | **0.46 (0.33–0.66) <0.001***** | **0.48 (0.33–0.68) <0.001***** | **0.47 (0.33–0.66) <0.001***** |
|  |  | Professional training (Yes) | 1.34 (0.99–1.80) 0.056 | 1.31 (0.97–1.77) 0.076 | **1.35 (1.00–1.81) 0.050*** |
|  |  | Safety education (Yes) | 0.93 (0.51–1.72) 0.828 | 0.93 (0.51–1.72) 0.820 | 0.94 (0.51–1.73) 0.845 |
|  |  | Binding test (Yes) | 1.35 (0.91–2.02) 0.140 | 1.35 (0.91–2.02) 0.140 | 1.36 (0.91–2.03) 0.134 |
|  |  | Warm-up: ≤ 10 min | 1.62 (0.70–3.75) 0.260 | 1.57 (0.68–3.63) 0.289 | 1.61 (0.70–3.72) 0.262 |
|  |  | Warm-up: 11-20 min | 1.46 (0.62–3.42) 0.387 | 1.42 (0.61–3.33) 0.415 | 1.47 (0.63–3.43) 0.377 |
|  |  | Warm-up: 21-30 min | 0.96 (0.38–2.38) 0.923 | 0.97 (0.39–2.41) 0.950 | 0.96 (0.39–2.39) 0.933 |
|  |  | Warm-up: > 30 min | 2.58 (0.92–7.21) 0.071 | 2.43 (0.87–6.79) 0.090 | 2.64 (0.95–7.36) 0.063 |
|  |  | Mood: excited | 1.10 (0.72–1.69) 0.657 | 1.15 (0.75–1.78) 0.525 | 1.09 (0.71–1.68) 0.682 |
|  |  | Mood: nervous | 1.20 (0.79–1.82) 0.399 | 1.25 (0.82–1.91) 0.300 | 1.20 (0.79–1.83) 0.386 |
|  |  | Mood: scared | 1.12 (0.72–1.75) 0.606 | 1.20 (0.77–1.87) 0.430 | 1.12 (0.72–1.74) 0.613 |
|  |  | Mood: low-spirited | 0.95 (0.50–1.83) 0.888 | 0.95 (0.50–1.84) 0.888 | 0.96 (0.50–1.84) 0.908 |
|  | **Experience Factors** | Skier level: Intermediate | 0.65 (0.35–1.20) 0.171 | 0.61 (0.33–1.13) 0.116 | 0.65 (0.35–1.21) 0.174 |
|  |  | Skier level: Advanced | **0.46 (0.23–0.90) 0.023*** | **0.42 (0.22–0.83) 0.012*** | **0.46 (0.23–0.90) 0.023*** |
|  |  | Experience: ≤ 1 season | 1.06 (0.66–1.70) 0.807 | 1.04 (0.65–1.67) 0.856 | 1.06 (0.66–1.69) 0.811 |
|  |  | Experience: 2-4 seasons | 1.19 (0.79–1.79) 0.413 | 1.17 (0.78–1.77) 0.445 | 1.19 (0.79–1.79) 0.394 |
|  |  | Experience: ≥ 5 seasons | 1.32 (0.83–2.10) 0.246 | 1.29 (0.81–2.07) 0.283 | 1.33 (0.83–2.11) 0.236 |
| **2. Demographics** |  | Sex (Female) | 0.88 (0.64–1.21) 0.419 | 0.60 (0.36–1.01) 0.052 | 0.90 (0.66–1.23) 0.495 |
| **3. Other Covariates** |  | Discipline (Snowboarding) | **0.67 (0.51–0.89) 0.005**** | **0.67 (0.51–0.89) 0.006**** | **0.67 (0.51–0.89) 0.005**** |
|  |  | Speed (Slow) | 0.90 (0.69–1.18) 0.458 | 0.90 (0.69–1.18) 0.445 | 0.90 (0.69–1.18) 0.454 |
|  |  | Injury mechanism: Collision with obstacles | **1.51 (1.09–2.09) 0.012*** | **1.47 (1.06–2.04) 0.020*** | **1.52 (1.10–2.10) 0.011*** |
|  |  | Injury mechanism: Collision with other participants | 1.18 (0.76–1.82) 0.467 | 1.17 (0.76–1.81) 0.484 | 1.16 (0.75–1.79) 0.498 |
|  |  | Injury mechanism: Backward fall | 1.08 (0.75–1.55) 0.678 | 1.10 (0.76–1.57) 0.616 | 1.07 (0.75–1.53) 0.713 |
|  |  | Location: Terrain parks | 0.81 (0.57–1.16) 0.257 | 0.81 (0.57–1.16) 0.251 | 0.82 (0.58–1.16) 0.266 |
|  |  | Weather: Overcast | 1.46 (0.85–2.51) 0.174 | 1.49 (0.86–2.59) 0.151 | 1.45 (0.85–2.48) 0.176 |
|  |  | Weather: Cloudy | 0.77 (0.56–1.06) 0.113 | 0.77 (0.56–1.07) 0.121 | 0.78 (0.57–1.06) 0.116 |
|  |  | Snow: Wet snow | 0.42 (0.13–1.34) 0.145 | 0.40 (0.13–1.29) 0.126 | 0.45 (0.14–1.39) 0.165 |
|  |  | Snow: Icy snow | 0.70 (0.35–1.42) 0.326 | 0.68 (0.33–1.38) 0.284 | 0.72 (0.36–1.45) 0.355 |
|  |  | Snow-sport musculoskeletal history (Yes) | 1.32 (0.97–1.79) 0.074 | **1.35 (1.00–1.83) 0.053** | 1.30 (0.96–1.76) 0.086 |
|  |  | Hypertension history (Yes) | 1.20 (0.84–1.72) 0.317 | 1.20 (0.83–1.72) 0.331 | 1.21 (0.85–1.73) 0.298 |
|  |  | Physical condition (Poor) | 0.31 (0.07–1.44) 0.136 | 0.31 (0.07–1.43) 0.133 | 0.31 (0.07–1.42) 0.132 |

Notes: Sensitivity 1: standard logistic regression with HC3 robust standard errors (Logit + HC3), corresponding to the fully adjusted covariate set. Sensitivity 2: BMI replaced by height and weight (both modeled using RCS; df = 4), with other adjustments unchanged. Sensitivity 3: knots for continuous variables (age, BMI, temperature, snow depth) reduced from df = 4 to df = 3 to assess model stability. Bold values indicate statistical significance (p < 0.05). **p* < 0.05; ***p* < 0.01; ****p* < 0.001. OR, odds ratio; CI, confidence interval; RCS, restricted cubic splines; BMI, body mass index; HC3, heteroskedasticity-consistent covariance matrix estimator 3.

**TABLE S2 Overall Wald tests for spline-modeled continuous variables in the fully adjusted model**

| **Variable** | **Model** | **Functional form** | **No. of spline terms** | **Overall Wald test *p* value** |
| --- | --- | --- | --- | --- |
| Age | Model 3 | Restricted cubic spline (df = 4) | 4 | 0.411 |
| BMI | Model 3 | Restricted cubic spline (df = 4) | 4 | 0.444 |
| Temperature | Model 3 | Restricted cubic spline (df = 4) | 4 | 0.257 |
| Snow depth | Model 3 | Restricted cubic spline (df = 4) | 4 | 0.010 |

Notes: BMI, body mass index. Overall *p* values were obtained from joint Wald tests of all spline basis terms for each variable in the fully adjusted Firth-penalized logistic regression model.

**TABLE S3 Tests for multiplicative interaction effects on severe snow-sport injury (fully adjusted Firth-penalized logistic regression model).**

| **Anchor variable** | **Counter variable** | **Interaction term** | **P value**  **(joint Wald test)** | **FDR-adjusted**  **(q = 0.10)** |
| --- | --- | --- | --- | --- |
| warmup duration | skiing experience | warmup duration × skiing experience | <0.001*** | Pass |
| warmup duration | risk behavior | warmup duration × risk behavior | <0.001*** | Pass |
| warmup duration | skier level | warmup duration × skier level | <0.001*** | Pass |
| binding test | skier level | binding test × skier level | <0.001*** | Pass |
| knee protector use | risk behavior | knee protector use × risk behavior | 0.001** | Pass |
| helmet use | discipline | helmet use × discipline | 0.049* | Fail |
| knee protector use | skier level | knee protector use × skier level | 0.055 | Fail |
| safety education | risk behavior | safety education × risk behavior | 0.082 | Fail |
| safety education | skier level | safety education × skier level | 0.115 | Fail |
| knee protector use | discipline | knee protector use × discipline | 0.146 | Fail |
| knee protector use | perceived speed | knee protector use × perceived speed | 0.170 | Fail |
| knee protector use | sex | knee protector use × sex | 0.212 | Fail |
| safety education | sex | safety education × sex | 0.240 | Fail |
| professional training | skier level | professional training × skier level | 0.254 | Fail |
| professional training | discipline | professional training × discipline | 0.377 | Fail |
| binding test | discipline | binding test × discipline | 0.378 | Fail |
| warmup duration | sex | warmup duration × sex | 0.414 | Fail |
| knee protector use | skiing experience | knee protector use × skiing experience | 0.426 | Fail |
| helmet use | sex | helmet use × sex | 0.447 | Fail |
| safety education | perceived speed | safety education × perceived speed | 0.458 | Fail |
| professional training | risk behavior | professional training × risk behavior | 0.467 | Fail |
| professional training | sex | professional training × sex | 0.468 | Fail |
| binding test | perceived speed | binding test × perceived speed | 0.478 | Fail |
| warmup duration | discipline | warmup duration × discipline | 0.545 | Fail |
| helmet use | risk behavior | helmet use × risk behavior | 0.551 | Fail |
| helmet use | perceived speed | helmet use × perceived speed | 0.601 | Fail |
| binding test | risk behavior | binding test × risk behavior | 0.607 | Fail |
| helmet use | skier level | helmet use × skier level | 0.646 | Fail |
| warmup duration | perceived speed | warmup duration × perceived speed | 0.725 | Fail |
| binding test | skiing experience | binding test × skiing experience | 0.762 | Fail |
| binding test | sex | binding test × sex | 0.768 | Fail |
| safety education | skiing experience | safety education × skiing experience | 0.813 | Fail |
| helmet use | skiing experience | helmet use × skiing experience | 0.869 | Fail |
| safety education | discipline | safety education × discipline | 0.878 | Fail |
| professional training | perceived speed | professional training × perceived speed | 0.879 | Fail |
| professional training | skiing experience | professional training × skiing experience | 0.931 | Fail |

Notes: Data were derived from systematic pairwise interaction testing in the fully adjusted model (corresponding to Model 3 in Table 2). Interactions were tested between selected core modifiable factors (anchor variables: knee protector use, helmet use, binding test, professional training, safety education, warm-up duration) and key covariates (counter variables: sex, discipline, perceived speed, risk behavior, skiing experience, skier level). Only interactions with nominal p < 0.25 are shown for brevity. Joint Wald test p values are reported. False discovery rate adjustment was applied across all tested interactions using the Benjamini–Hochberg procedure (*q* = 0.10). “Pass” indicates significance after FDR correction. **p* < 0.05; ***p* < 0.01; ****p* < 0.001. FDR, false discovery rate.

**TABLE S4 Selected exposure-intervention combinations: joint effects on severe snow-sport injury (fully adjusted Firth-penalized logistic regression models).**

| **Interaction** | **Combination** | **OR (95% CI)** | **P value** |
| --- | --- | --- | --- |
| **Main Joint Effects** |  |  |  |
| Perceived speed × Knee protector use | fast × no | 1.000 (Reference) | - |
|  | fast × yes | 0.497 (0.045–7.267) | 0.138 |
|  | slow × no | 0.601 (0.028–10.523) | 0.162 |
|  | slow × yes | 0.480 (0.046–8.310) | 0.134 |
| Perceived speed × Professional training | fast × no | 1.000 (Reference) | - |
|  | fast × yes | 1.281 (0.174–4.410) | 0.162 |
|  | slow × no | 0.890 (0.254–2.745) | 0.118 |
|  | slow × yes | 1.184 (0.227–4.470) | 0.151 |
| Risk behavior × Helmet use | more risk-taking × no | 1.000 (Reference) | - |
|  | more risk-taking × yes | 1.319 (0.030–2203.456) | 0.232 |
|  | more cautious × no | 0.786 (0.012–369.534) | 0.153 |
|  | more cautious × yes | 0.634 (0.030–279.230) | 0.127 |
| Risk behavior × Safety education | more risk-taking × no | 1.000 (Reference) | - |
|  | more risk-taking × yes | 0.407 (0.000–42504.570) | 0.220 |
|  | more cautious × no | 0.176 (0.000–70709.130) | 0.109 |
|  | more cautious × yes | 0.215 (0.001–104650.200) | 0.129 |
| **Supplementary Joint Effects** | | | |
| Perceived speed × Warm-up duration | fast × no warm-up | 1.000 (Reference) | - |
|  | fast × ≤10 min | 1.849 (0.196–121.509) | 0.154 |
|  | fast × 11-20 min | 1.943 (0.149–143.780) | 0.160 |
|  | fast × 21-30 min | 1.147 (0.059–149.810) | 0.101 |
|  | fast × >30 min | 3.202 (0.005–650.600) | 0.239 |
|  | slow × no warm-up | 1.448 (0.036–89.321) | 0.125 |
|  | slow × ≤10 min | 1.905 (0.218–105.288) | 0.158 |
|  | slow × 11-20 min | 1.519 (0.183–70.360) | 0.130 |
|  | slow × 21-30 min | 1.246 (0.067–165.373) | 0.109 |
|  | slow × >30 min | 2.158 (0.000–135.464) | 0.175 |
| Risk behavior × Warm-up duration | more risk-taking × no warm-up | 1.000 (Reference) | - |
|  | more risk-taking × ≤10 min | 7656316.000 (1.000–33763050.000) | 0.008 |
|  | more risk-taking × 11-20 min | 682528400.000 (1.000–10888760.000) | 0.406 |
|  | more risk-taking × 21-30 min | 1267963.000 (1.000–2869866.000) | 0.001 |
|  | more risk-taking × >30 min | 1343225000.000 (1.000–317334400000.000) | 0.573 |
|  | more cautious × no warm-up | 86198990.000 (1.000–729925200.000) | 0.079 |
|  | more cautious × ≤10 min | 46840480.000 (2.226–704645100.000) | 0.045 |
|  | more cautious × 11-20 min | 226825500.000 (3.433–551512200.000) | 0.185 |
|  | more cautious × 21-30 min | 10234540.000 (1.000–600009000.000) | 0.010 |
|  | more cautious × >30 min | 308839800.000 (1.000–10585840000.000) | 0.236 |

Notes: OR, odds ratio. CI, confidence interval.


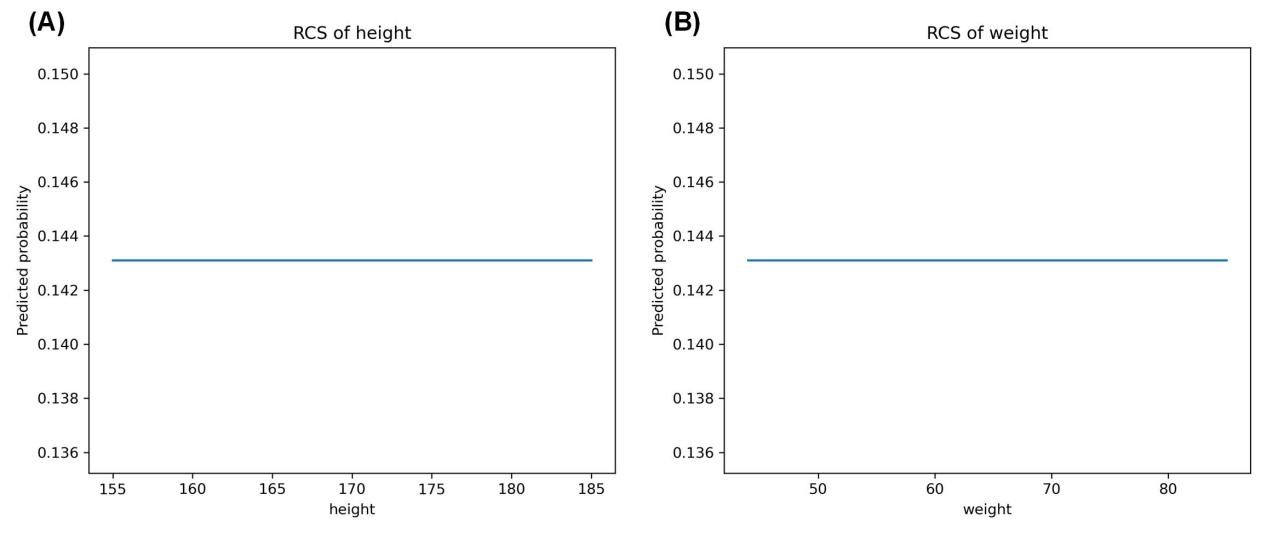


**FIGURE S1 Nonlinear associations of height and weight with severe snow-sport injury using restricted cubic splines.**

Notes: RCS, restricted cubic splines. (A), RCS of height. (B), RCS of weight.


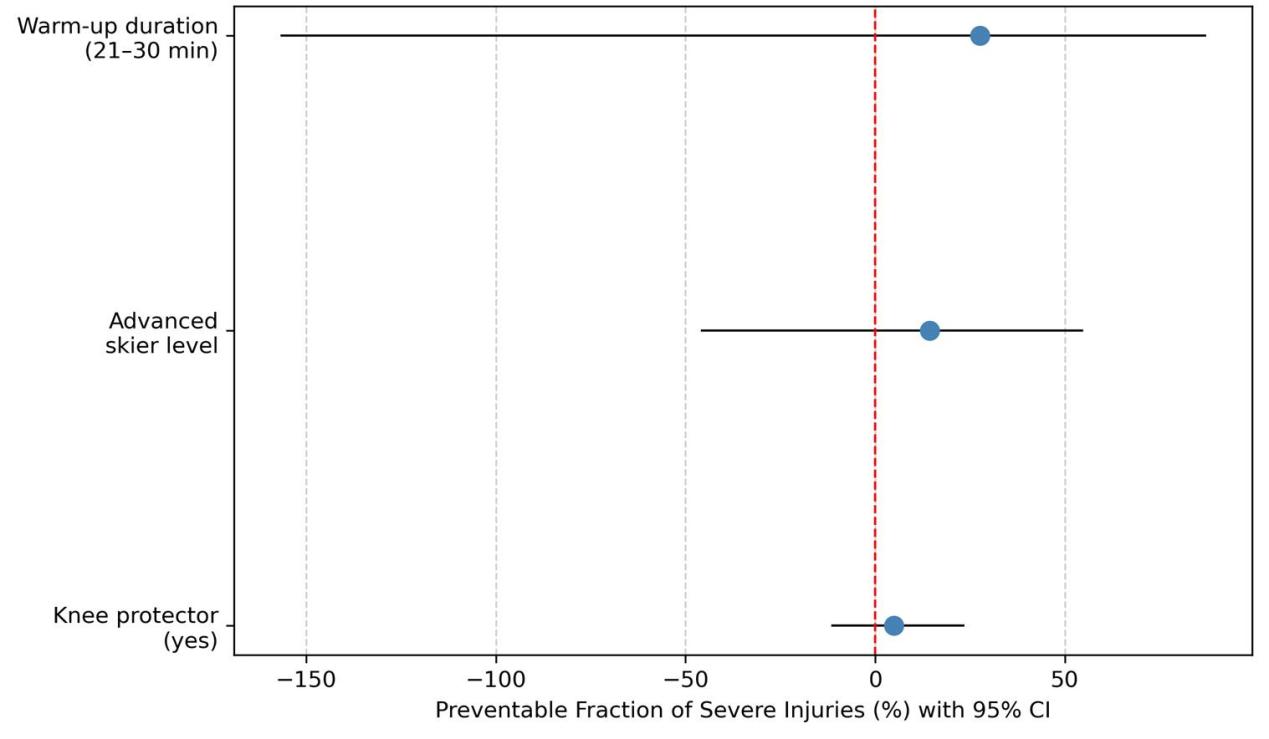


**FIGURE S2 Population fraction preventable by modifiable factor scenario with 95% confidence intervals.**

Notes: PFP, population fraction preventable (standardized to the injured cohort) = (baseline predicted risk − counterfactual predicted risk under the optimal level)/baseline predicted risk, with risks averaged over the analytic population of injured participants. CI, confidence interval.
